# *Spirulina* Lipids Alleviate Oxidative Stress and Inflammation in Mice Fed a High-Fat and High-Sucrose Diet

**DOI:** 10.3390/md18030148

**Published:** 2020-03-04

**Authors:** Yuhong Yang, Lei Du, Masashi Hosokawa, Kazuo Miyashita

**Affiliations:** 1 College of Food Science and Engineering, Qilu University of Technology, No.3501, Daxue Road, Jinan 250353, China; yyhforever1108@163.com; 2 Department of Nutrition and Food Hygiene, School of Public Health, Shandong University, No.44 Wenhuaxi Road, Jinan 250012, China; 3 Faculty of Fisheries Sciences, Hokkaido University, 3-1-1 Minato-cho, Hakodate, Hokkaido 041-8611, Japan

**Keywords:** *Spirulina* lipids, gamma-linolenic acid, carotenoids, oxidative stress, inflammation

## Abstract

High-fat and high-sucrose diet (HFHSD)-induced obesity leads to oxidative stress and chronic inflammatory status. However, little is known about the beneficial effects of total lipids extracted from *Spirulina*. Hence, in the present study, *Spirulina* lipids were extracted with chloroform/methanol (SLC) or ethanol (SLE) and then their effects on oxidative stress and inflammation in the mice fed a HFHSD were investigated. The results show that the major lipid classes and fatty acid profiles of SLC and SLE were almost similar, but the gamma-linolenic acid (GLA) and carotenoid contents in SLE was a little higher than that in SLC. Dietary 4% SLC or SLE for 12 weeks effectively decreased the hepatic lipid hydroperoxide levels as well as increased the activities and mRNA levels of antioxidant enzymes in the mice fed a HFHSD. In addition, supplementation with SLC and SLE also markedly decreased the levels of serum pro-inflammatory cytokines and the mRNA expression of pro-inflammatory cytokines in the liver and epididymal white adipose tissue of mice fed a HFHSD, and the effects of SLC and SLE were comparable. These findings confirm for the first time that dietary *Spirulina* lipids could alleviate HFHSD-induced oxidative stress and inflammation.

## 1. Introduction

Oxidative stress refers to an imbalance between excessive productions of reactive oxygen species (ROS) and their elimination by protective mechanisms [1]. Evidence suggests that oxidative stress is a critical factor for pathological obesity, which is considered a serious health burden worldwide because of its associated complications, such as diabetes, cardiovascular diseases, and hepatic steatosis [2,3]. It has been reported that ROS are selectively increased in the adipose tissue of obese individuals, together with the increase in nicotinamide adenine dinucleotide phosphate (NADPH) oxidase activity and the downregulation of antioxidative enzyme expression [4]. High-calorie diet induced obesity can induce systemic oxidative stress through various mechanisms, including superoxide generation from the NADPH oxidases, oxidative phosphorylation, glyceraldehyde auto-oxidation, protein kinase C activation, polyol, and hexosamine pathways [5]. More recently, studies have also suggested that oxidative stress is a risk factor for premature atherosclerosis, diabetes, and hypertension [6,7,8].

It has been reported that many inflammation and macrophage-specific genes are dramatically upregulated in the white adipose tissue (WAT) of genetic and high-fat diet-induced obese mouse models [9]. Other studies have also demonstrated that the development of obesity increases the secretions of various pro-inflammatory chemokines and cytokines from adipose tissues, such as tumor necrosis factor-alpha (TNF-α), interleukin-1beta (IL-1β), IL-6 and monocyte chemoattractant protein-1 (MCP-1) [10,11,12]. MCP-1 recruits monocytes to the adipose tissue, causing a chronic low-grade inflammation characterized by the overproduction of pro-inflammatory molecules and the downregulation of an anti-inflammatory adiponectin [9,13,14]. During obesity development, pro-inflammatory cytokines, such as TNF-α,derived from adipocytes and macrophages, organize a paracrine loop that leads to inflammation in the adipose tissue and then increase the secretion of more pro-inflammatory molecules [15]. The increase in the secretion of pro-inflammatory cytokines from the adipose tissue has been reported to promote insulin resistance [16,17,18]. Increasing evidence from human studies and animal research have established correlative links between chronic inflammation in adipose tissue and insulin resistance [9].

In contrast, the expression of inflammatory-inducing molecules is known to be upregulated by the oxidative stress increase in obese individuals, and this inflammation is implicated in the development of various metabolic disorders, such as insulin resistance [4,9,19,20,21,22]. Therefore, the reduction in oxidative stress and the increase in endogenous antioxidant defenses may be effective at improving obesity-associated metabolic dysfunctions [23,24]. From this viewpoint, much attention has been paid to antioxidant-rich food products [6,21,23,24]. Furthermore, some of the antioxidants in these products are interesting because of their anti-obesity activity through different molecular mechanisms, such as direct stimulation of energy expenditure [25,26,27,28].

*Spirulina* (*Arthrospira platensis*) is a filamentous blue-green microalgae used as a nutraceutical food supplement due to its high content in protein, essential fatty acids, vitamins, polyphenols, and carotenoids [29]. Recently, many studies focus on the potential antioxidant activity of *Spirulina* since its antioxidant, anti-inflammatory, and hypolipidemic properties were demonstrated in a large number of preclinical studies, showing the great benefits of algae against conditions including hypercholesterolemia, hyperglyceridemia, cardiovascular diseases, and inflammatory diseases [30,31]. *Spirulina* contains several hydrophilic active ingredients, such as phycocyanin and phycocyanobilin. These water-soluble pigments show promising antioxidant, immunomodulatory and anti-inflammatory properties [30,31,32]. Recent studies also show that *Spirulina* lipids are major factors responsible for health beneficial effects [33,34,35,36]. Coué et al. reported that the liquid *Spirulina* extract effectively attenuates hepatic fibrosis, inflammation, oxidative stress, and insulin resistance in C57Bl/6J mice fed a western-style diet [37]. The main polyunsaturated fatty acids (PUFAs) of *Spirulina* lipids is gamma-linolenic acid (GLA, C18:3*n*-6). Generally, eicosapentaenoic acid (EPA, C20:5*n*-3) and docosahexaenoic acid (DHA, C22:6*n*-3) play a positive role in the amelioration of obesity and diet-associated metabolic syndromes such as insulin resistance and dyslipidemia [38]. However, GLA feeding has significantly reduced WAT weight of rats fed with a high-fat diet [39]. Fatty acids including GLA in *Spirulina* lipids are mainly present as glycolipids (GLs), such as monogalactosyl diacylglycerol (MGDG), digalactosyl diacylglycerol (DGDG), and sulfoquinovosyl diacylglycerol (SQDG). These GLs have been reported to inhibit fat accumulation in adipose cells [40,41]. Moreover, our previous study showed that supplementation with 4% *Spirulina* lipids could alleviate high-fat and high-sucrose diet (HFHSD)-induced obesity and hepatic lipid accumulation [42]. However, the antioxidant and anti-inflammatory activities of *Spirulina* lipids are still unknown, although they contain several antioxidants, such as carotenoids.

The current study aims to investigate the effects of *Spirulina* lipids on oxidative stress and chronic inflammatory status in mice fed a HFHSD. Several studies indicate that the extraction solvents significantly affect the content and effectiveness of the extracted lipid compounds [43,44]. Hence, in the present study, the total lipids were extracted from *Spirulina* with chloroform/methanol (2:1, v/v) or ethanol, namely, the *Spirulina* lipids extracted with chloroform/methanol (SLC) and the *Spirulina* lipids extracted with ethanol (SLE), and then the antioxidant and anti-inflammatory effects of both lipid extracts were evaluated.

## 2. Results

### 2.1. Chloroform/Methanol (SLC) and Ethanol (SLE) Analysis and Fatty Acid Composition of Dietary Lipids

The thin-layer chromatography (TLC) analysis showed that the major lipid classes of SLC and SLE were GLs and phospholipids (PLs). SLC contained 52% GLs and 26% PLs, while the GLs and PLs contents in SLE were 46% and 30%, respectively. The present results also reveal that the major GLs’ constituents in SLC and SLE were MGDG, DGDG, and SQDG, whereas the main components of PLs were phosphatidylethanolamine and phosphatidylglycerol.

The ingredient compositions of the experimental diets are shown in Table 1. Soybean oil and lard were used as basic dietary lipids in the present study. The fatty acid composition of *Spirulina* lipids and the dietary lipids are shown in Table 2. The fatty acid profiles of SLC and SLE were almost similar, although the extraction solvents were different. Both SLC and SLE were mainly composed of palmitic acid, linoleic acid (LA), and GLA. The higher level of the characteristic PUFAs of *Spirulina* lipids, GLA, was 22.06% in SLE, whereas it was 20.28% in SLC. The major fatty acids of dietary lipids were palmitic acid, stearic acid, oleic acid, and LA, which were mainly originated from soybean oil and lard. GLA was only found in the diet containing SLC or SLE.

The carotenoid contents of SLC and SLE are shown in Table 3. SLC contained 9.29 mg/g lipids of β-carotene and 6.18 mg/g lipids of zeaxanthin, while SLE presented 8.57 mg/g lipids and 8.69 mg/g lipids of β-carotene and zeaxanthin, respectively.

Overall, the present results indicate that the major lipid classes and fatty acid profiles of SLC and SLE were almost similar, but the GLA and carotenoid contents in SLE was a little higher than that in SLC.

### 2.2. Lipid Hydroperoxide Levels in the Liver

**Figure 1:**
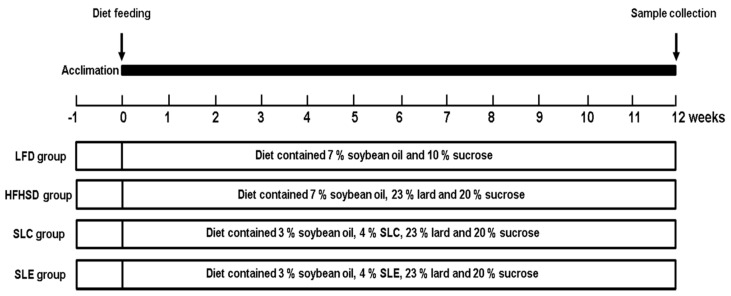
A flow chart of the present study design.

**Figure 2:**
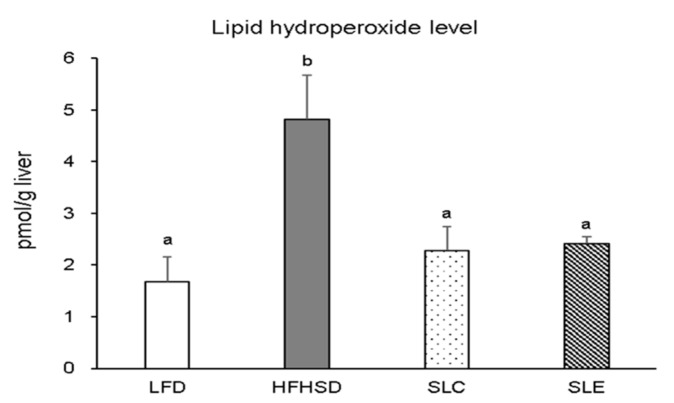
Lipid hydroperoxide level in the liver of mice fed experimental diets. The values are expressed as mean ± SE (*n* = 7) and different letters indicate a significant difference at *P* < 0.05.

A flow chart of the study design is presented in Figure 1. Firstly, the lipid hydroperoxide levels in the livers of mice were investigated. As shown in Figure 2, the hepatic lipid hydroperoxide levels in mice fed a HFHSD were significantly higher than that of mice fed an LFD. In contrast, supplementation with 4% SLC or SLE significantly reduced the lipid hydroperoxide levels in the liver of mice as compared to the mice fed a HFHSD, while no significant difference was observed between SLC and SLE groups.

### 2.3. Antioxidant Enzyme Activity and GSSG/GSH Ratio in the Liver and Epididymal WAT

**Figure 3:**
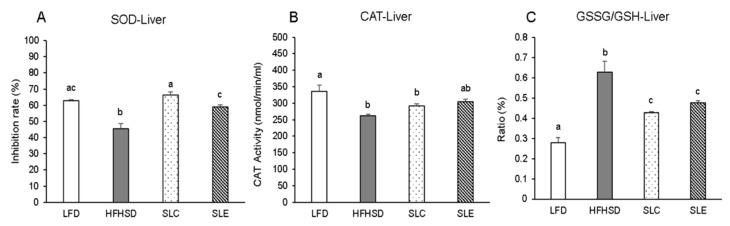
Enzyme activities of the superoxide dismutase (SOD) (**A**), catalase (CAT) (**B**), and GSSG/GSH (**C**) ratios in the liver of mice fed experimental diets. The values are expressed as mean ± SE (*n* = 7) and different letters indicate a significant difference at *P* < 0.05.

**Figure 4:**
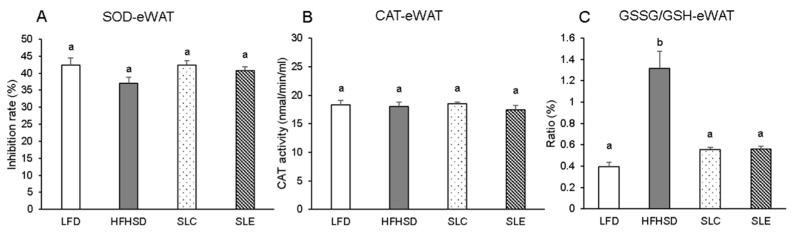
Enzyme activities of SOD (**A**), CAT (**B**), and GSSG/GSH (**C**) ratios in the eWAT of mice fed experimental diets. The values are expressed as mean ± SE (*n* = 7) and different letters indicate a significant difference at *P* < 0.05.

The activities of antioxidant enzymes were measured in the liver (Figure 3) and epididymal WAT (eWAT) (Figure 4). Superoxide dismutase (SOD) activity significantly decreased in the liver of mice fed a HFHSD as compared to the LFD group (Figure 3A). The hepatic SOD activity markedly increased with SLC or SLE administration to the same level as in the LFD group, and the effect of SLC was superior to SLE. The hepatic CAT activity was also significantly lower in HFHSD-fed mice than that of in LFD group (Figure 3B). SLC or SLE supplementation increased the hepatic catalase (CAT) activity, but there was no significant difference among the HFHSD, SLC, and SLE groups (Figure 3B). The ratio of reduced oxidized glutathione (GSSG)/glutathione (GSH) in the liver was significantly increased with HFHSD feeding as compared to the LFD group. However, the ratio was dramatically lowered with SLC or SLE supplementation (Figure 3C). In addition, the similar effect of SLC or SLE administration was also observed on the GSSG/GSH ratio in the eWAT of mice (Figure 4C). However, there were no significant differences in SOD and CAT activities of eWAT among the mice fed experimental diets (Figure 4A,B).

### 2.4. mRNA Expression of Antioxidant Enzymes in the Liver and eWAT.

**Figure 5:**
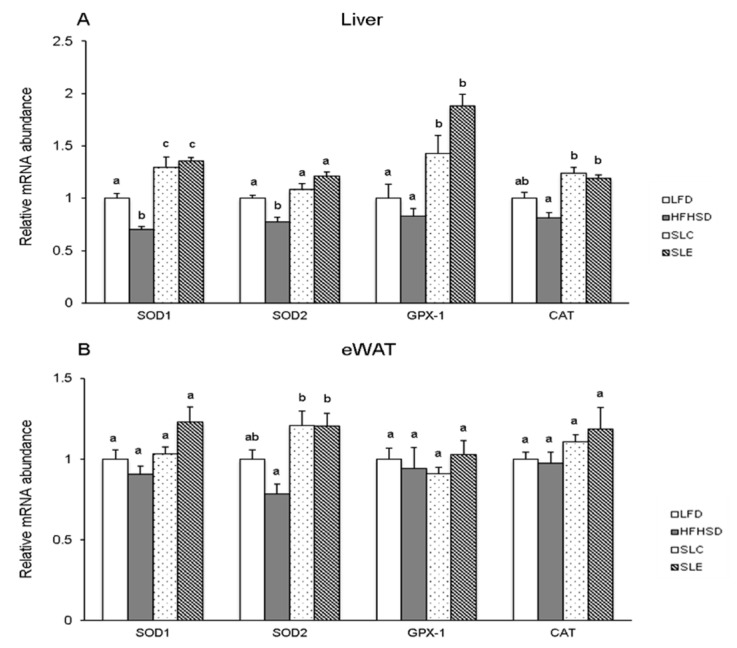
Relative mRNA expression of antioxidant enzymes in the liver (**A**) and the eWAT (**B**) of mice fed experimental diets. Data normalization was accomplished using the endogenous reference GAPDH. The values are expressed as mean ± SE (*n* = 7) and different letters indicate a significant difference at *P* < 0.05.

To determine the effects of dietary *Spirulina* lipids on antioxidant enzymatic activities, related mRNA expression in the liver and eWAT were analyzed by qRT-PCR. The analysis showed the reduction of hepatic mRNA expression of antioxidant enzymes such as SOD1 (CuZn-SOD), SOD2 (Mn-SOD), glutathione peroxidase 1 (GPX-1) and CAT in HFHSD-fed mice as compared to the LFD group (Figure 5A). Among them, significant differences were observed in the mRNA expression of SOD1 and SOD2. The results also demonstrate that SLC or SLE supplementation significantly increased the hepatic mRNA expression of all antioxidant enzymes as compared to the HFHSD group. Furthermore, the mRNA expression of SOD1 and GPX-1 in the liver of mice fed SLC or SLE were significantly higher than that of mice fed an LFD (Figure 5A). In contrast, there was no significant difference in the eWAT mRNA expression of antioxidant enzymes between the LFD and HFHSD groups, while a significant increase was observed in SOD2 mRNA level of mice fed SLC or SLE as compared to the HFHSD group (Figure 5B).

### 2.5. Serum Pro-Inflammatory Cytokines Levels

**Figure 6:**
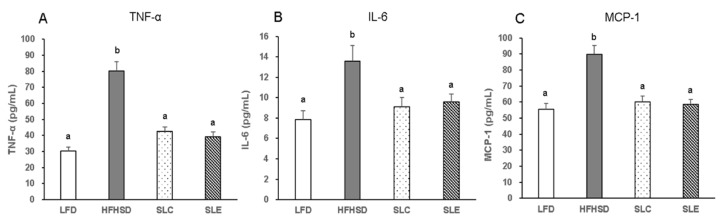
Serum pro-inflammatory cytokines’ levels in the mice fed experimental diets. **A**: TNF-α; **B**: IL-6; **C**: MCP-1. The values are expressed as mean ± SE (*n* = 7) and different letters indicate a significant difference at *P* < 0.05.

Serum pro-inflammatory cytokines levels are shown in Figure 6. The TNF-α, IL-6, and MCP-1 levels were significantly higher in the HFHSD group as compared to the mice fed an LFD. However, dietary SLC or SLE inhibited the increase of these inflammatory cytokines notably in the serum of mice fed a HFHSD. In addition, the lowering effects of SLC and SLE on serum pro-inflammatory cytokines’ levels were comparable.

### 2.6. mRNA Expression of Pro-Inflammatory Cytokines in the Liver and eWAT

**Figure 7:**
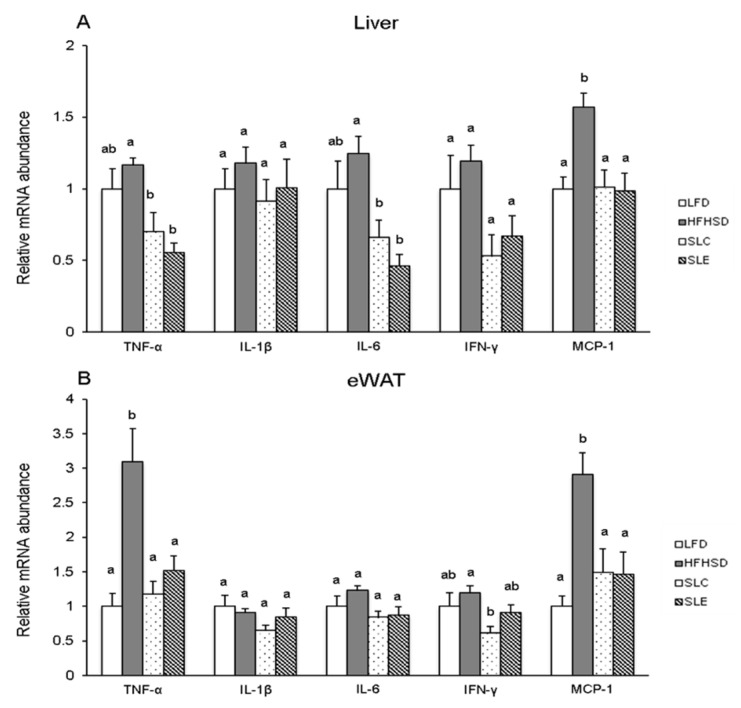
Relative mRNA expression of pro-inflammatory cytokines in the liver (**A**) and the eWAT (**B**) of mice fed experimental diets. Data normalization was accomplished using the endogenous reference GAPDH. The values are expressed as mean ± SE (*n* = 7) and different letters indicate a significant difference at *P* < 0.05.

As for the mRNA expression of pro-inflammatory cytokine in the liver (Figure 7A), the increase of these cytokine mRNA levels was found in mice fed a HFHSD when compared to LFD-fed ones, although the significant difference was only observed on hepatic MCP-1 mRNA expression. On the contrary, dietary SLC or SLE reduced the mRNA expression of pro-inflammatory cytokines as compared to the expression in the HFHSD group. In particular, significant decreases were observed in TNF-α, IL-6, and MCP-1 mRNA levels in the liver. In addition, a similar effect was also found in the eWAT of mice fed SLC or SLE (Figure 7B). The present results also reveal that SLC supplementation decreased the mRNA expression of TNF-α, INF-γ, and MCP-1 significantly in the eWAT of mice fed a HFHSD, whereas the decrease was notable in TNF-α and MCP-1 mRNA levels in SLE group.

### 2.7. Carotenoids Accumulation in the Liver, eWAT, and Small Intestine

SLC and SLE contained β-carotene and zeaxanthin as major carotenoids (Table 3). These carotenoids were found in the small intestine but were not detected in the liver or the eWAT of mice fed SLC or SLE. Zeaxanthin content was little higher than β-carotene in the small intestine of mice fed *Spirulina* lipids. Because SLE contained a lower level of β-carotene and a higher level of zeaxanthin than that in SLC, similar carotenoid levels in the small intestine were observed in these two groups (Table 3).

## 3. Discussion

The present study reveals the significant increase in hepatic lipid hydroperoxide levels in the HFHSD group and the remarkable reduction in hydroperoxide levels with SLC or SLE supplementation (Figure 2). Our previous study found that the most abundant PUFAs among the hepatic lipids in mice fed an LFD or *Spirulina* lipids were LA (C18:2*n*-6), arachidonic acid (C20:4*n*-6, AA), and DHA [42]. Among the PUFAs, AA, and DHA are major targets in lipid oxidation because both PUFAs are very easily oxidized in model systems as the oxidative stability of each PUFA is inversely proportional to its degree of unsaturation [45,46]. Considering the AA content, the hepatic lipids of mice fed an LFD, SLC, and SLE were expected to be more easily oxidized than the mice fed a HFHSD. However, as shown in Figure 2, the hepatic lipid hydroperoxide levels of the LFD, SLC, and SLE groups were significantly lower than that of the HFHSD group. The main lipid classes and PUFAs in *Spirulina* lipids are GLs and GLA, respectively. In addition, both *Spirulina* lipid extracts contained β-carotene and zeaxanthin as major antioxidants. Hence, the lowering effect of *Spirulina* lipids on hepatic lipid hydroperoxides levels would be due to their characteristic lipid compositions and liposoluble components.

Evidence shows that the first line of defense against ROS in biological systems consists of antioxidant enzymes such as SOD and CAT, which inactivate and/or scavenge ROS [47]. SOD is the only enzyme that disrupts superoxide radicals, and CAT prevents the formation of hydroxyl radical abolishing H_2_O_2_ produced by free radicals or by SOD reaction [47,48]. The activities of both enzymes were significantly reduced in the liver of HFHSD-fed mice as compared to the LFD group (Figure 3A,B). Reduced activity of these hepatic antioxidants resulted in the accumulation of ROS in the liver of the HFHSD group [49] and then the lipid hydroperoxide levels would increase. SLC or SLE supplementation improved these antioxidant enzymatic activities and prevented the lipid oxidation.

The increase in the antioxidant defense in the livers of mice fed SLC or SLE is evident from the analysis of the GSSG/GSH ratio (Figure 3C). GSH serves as an electron donor and it very easily reacts with ROS converting into its oxidized form, GSSG [50]. GSSG can be reduced to GSH through glutathione reductase in the presence of reduced NADPH [51]. Therefore, the quantitative determination of the GSSG/GSH ratio is accepted as a marker of the oxidative stress status in tissues [52]. The GSSG/GSH ratio in the liver and the eWAT of mice fed a HFHSD were significantly higher than those of the LFD group (Figure 3C and 4C). The ratio significantly decreased, replacing part of the soybean oil with SLC or SLE in the HFHSD group, probably due to the reduction in the oxidative stress induced by the higher levels of ROS in this group. The upregulation of the biological defense against oxidative stress through SLC or SLE is further supported by the increase in hepatic mRNA expression of SOD1, SOD2, GPX-1, and CAT (Figure 5A) as well as on SOD2 mRNA levels in the eWAT of mice (Figure 5B).

Excess accumulation of lipids in abdominal WAT is known to reduce its antioxidant ability and to increase ROS production [4]. Increased oxidative stress has also been found in fatty liver [53]. Our previous findings revealed that significant increase in hepatic weight and lipid content was found in the mice fed a HFHSD as compared to an LFD and *Spirulina* lipid groups [42]. Hence, higher oxidation levels in the liver of mice fed a HFHSD may be strongly related to their high hepatic lipid levels. Although the experimental diet of SLC and SLE groups had the same lipid and sucrose contents as the HFHSD group, replacing part of soybean oil with SLC or SLE in HFHSD increased the defense ability against ROS attack and decreased the lipid hydroperoxide to the same level as in LFD group.

Takahashi et al. reported that GLA caused significantly less body fat accumulation in rats fed a high-fat diet [39]. Several studies reported the inhibitory effect of GLs on fat accumulation in adipose cells [40,41]. Hence, among the lipid components of *Spirulina* lipids, GLA bonded to GLs may possibly be the main contributor reducing the excess fat accumulation in the liver induced by the HFHSD. Furthermore, several studies have reported that GLA exerts a potent anti-inflammatory activity both in vitro and in vivo [54,55,56]. Oxidative stress causes several types of pathological states including inflammation. And the secretion of pro-inflammatory cytokines is upregulated, resulting in the increase in ROS production [57]. Therefore, the anti-inflammatory effect of GLA would be strongly related to the decrease in hepatic oxidative stress caused by HFHSD-induced hepatic lipid accumulation.

In the obese state, enlarged adipocytes result in the infiltration of macrophages and the unbalance of pro-inflammatory and anti-inflammatory factors secreted by adipose tissue, which leads to the promotion of inflammation [58]. Chronic inflammation mediated by HFHSD-induced obesity is marked by increased pro-inflammatory cytokines such as TNF-α, IL-1β, and IL-6 in circulation [59]. The present findings demonstrate that supplementation with SLC or SLE blocked the increase in TNF-α, IL-6, and MCP-1 dramatically in the serum of mice fed a HFHSD (Figure 6). Moreover, the hepatic mRNA levels of TNF-α, IL-6, and MCP-1 were markedly decreased with SLC or SLE supplementation (Figure 7A). The significant decreases in TNF-α and MCP-1 mRNA expression were also found in the eWAT of mice fed SLC or SLE, as compared to the HFHSD group (Figure 7B). These decreases in pro-inflammatory cytokines would be partly due to the GLA as the major PUFAs in SLC and SLE.

Carotenoids are also important candidates explaining the antioxidant and anti-inflammatory activities of *Spirulina* lipids found in the present study. Several studies have reported that β-carotene is a potent antioxidant with increase in SOD activity and other antioxidant enzymes in a murine model on oxidative stress status [60,61,62]. Other studies showed that β-carotene inhibited the production of nitric oxide and prostaglandin E2 as well as suppressed the expression of inducible nitric oxide synthase, cyclooxygenase-2, TNF-α, and IL-1β both in vitro and in vivo [63]. Therefore, the β-carotene and zeaxanthin contents were measured in the liver, eWAT, and small intestine of mice fed SLC or SLE. The β-carotene and zeaxanthin were found in the small intestine of mice fed SLC or SLE. However, both carotenoids were not detected in the liver and eWAT (Table 3). To make clear the involvement of *Spirulina* carotenoids in the antioxidant and anti-inflammatory properties of SLC and SLE, further research is needed to optimize the method and conditions to define the *Spirulina* carotenoids content in the liver, WAT, and other organs or tissues.

## 4. Materials and Methods

### 4.1. Materials

*Spirulina* was provided by DIC Corporation (Tokyo, Japan). Soybean oil and lard were purchased from Wako Pure Chemical Ind. Ltd. (Osaka, Japan) and Junsei Chemical Co. Ltd. (Tokyo, Japan), respectively. All other chemicals and solvents used in the present study were analytical grade.

### 4.2. Preparation of Spirulina lipids

The raw *Spirulina* was dried and powdered firstly. Then, the *Spirulina* powder was extracted with chloroform/methanol (2:1, v/v) or ethanol overnight under dark conditions. The organic solvents were collected via filtration and concentrated in a rotary to obtain the *Spirulina* extracts. Chloroform/methanol/water (1:1:0.9, v/v/v) was added into the *Spirulina* extracts in a separatory funnel to remove water-soluble components. After shaking the funnel vigorously, the lower layer containing lipids was evaporated under reduced pressure in a rotary evaporator to obtain SLC and SLE.

### 4.3. Analysis of SLC and SLE

The lipid class profiles of SLC and SLE were analyzed using TLC. Each lipid sample was dissolved in a chloroform/methanol/water solution (65:25:4, v/v/v) and was spotted onto 0.25 mm silica gel 60 F254 plates (Merck, Darmstadt, Germany). The plates were developed with a chloroform/methanol/water (65:25:4, v/v/v) mixture and then the spots were visualized by spraying and charring the plates with orcinol-sulfuric acid or Dittmer reagent to detect GLs and PLs, respectively. Then, to analyze the non-polar lipids of SLC and SLE, *n*-hexane/diethyl ether/acetic acid (70:30:1, v/v/v) was used as a developing solvent. The spot was detected using 50% aqueous sulfuric acid charring. The lipid class compositions of SLC and SLE were identified by co-chromatography with pure standards (Sigma-Aldrich, St. Louis, MO, USA).

The fatty acid methyl esters (FAME) of SLC and SLE were prepared according to the method described by Prevot and Mordret [64]. Briefly, the lipids (*ca*. 10 mg) were dissolved in 1 mL of *n*-hexane and 0.2 mL of 2 N NaOH in methanol and were then vortexed and incubated at 50 °C for 20 to 30 s. After incubation, 0.2 mL of 2 N HCl in methanol solution was added and vortexed. The mixture was separated by centrifugation at 3000 rpm for 5 min. Then, the upper *n*-hexane layer containing FAME was recovered and subjected to gas chromatography (GC). The GC analysis was performed using a Shimadzu GC-14B (Shimadzu Seisakusho Ltd., Kyoto, Japan) equipped with a flame-ionization detector and a capillary column (Omegawax-320; 30 m × 0.32 mm i.d.; Supelco Inc., Bellefonte, PA, USA). The detector, injector and column temperatures were 260, 250, and 200 °C, respectively. The carrier gas was helium at a 50 kPa pressure. The fatty acid contents of *Spirulina* lipids were expressed as a weight percentage of the total fatty acids by comparing the retention times with a FAME standard mixture (Supelco Inc., Bellefonte, PA, USA).

The carotenoid profiles of *Spirulina* lipids were identified by high performance liquid chromatography (HPLC) using a Hitachi L-7000 HPLC system (Hitachi Seisakusho, Co., Tokyo, Japan) equipped with a pump (L-7100), an auto-sampler (L-7200), a photodiode array detector (L-7455), and an online analysis software (Model D-7000). The analysis was carried out at 25 °C using two connected reversed-phase columns (Develosil C30-UG-5, 250 × 4.6 mm i.d.; Nomura Chem. Co., Seto, Aichi, Japan) protected with a guard column with the same stationary phase. HPLC separation was carried out at a flow rate of 1.0 mL/min. Carotenoid elution was achieved using the following linear gradient: start condition, 100% A and 0% B; 20 min, 100% A and 0% B; 40 min, 50% A and 50% B; 60 min, 50% A and 50% B. A was methanol and B was dichloromethane. The injection volume of each sample was 30 µL. Carotenoids were detected at 250–700 nm and identified based on their retention times and UV/Vis absorption spectra, compared to the retention times of the carotenoid standards. Carotenoid quantification was determined using a standard curve based on commercial β-carotene and zeaxanthin (Wako Pure Chemical Industries Ltd., Osaka, Japan).

### 4.4. Animals and Diets

All procedures for the use and care of animals for this study were performed under the approval of the Ethical Committee of Experimental Animal Care at Hokkaido University. C57BL/6J male mice (4 weeks old, *n* = 28) were purchased from Charles River Laboratories Inc. (Yokohama, Kanagawa, Japan). All mice were housed at a constant temperature of 23 ± 1 °C and 50% humidity with a 12 h light–dark cycle throughout the experiment. After acclimation for a week, mice were randomly divided into four groups (*n* = 7/group) and fed the experimental diets based on the recommendations of the standard American Institute of Nutrition-93G (AIN-93G) diets [65] for a further 12 weeks. The ingredient compositions of the experimental diets are shown in Table 1. A flow chart of the study design is presented in Figure 1. The different groups include the low fat diet (LFD) group (contained 7% soybean oil and 10% sucrose), the HFHSD group (contained 7% soybean oil, 23% lard and 20% sucrose), the SLC group (contained 3% soybean oil, 4% SLC, 23% lard and 20% sucrose), and the SLE group (contained 3% soybean oil, 4% SLE, 23% lard and 20% sucrose). The total lipids of experimental diets were extracted and subjected to GC.

After 12 weeks of feeding, the mice were euthanized after overnight fasting. The blood was collected and then the serum was separated from whole blood by centrifugation at 4000 rpm for 15 min and stored at −80 °C. After blood collection, the liver, eWAT and small intestine were rapidly excised and weighted. All the organs and tissues were snap-frozen in liquid nitrogen and stored at −80 °C or RNAlater solution (Invitrogen, Carlsbad, CA, USA) until further analysis.

### 4.5. Hydroperoxide Analysis of Hepatic Lipids

The hydroperoxide content of hepatic lipids were determined through HPLC based on the quantitative conversion of non-fluorescent diphenyl-1-pyrenylphosphine (DPPP) to fluorescent DPPP oxide [66]. In brief, 10 mg of the hepatic lipid extract was dissolved in 5 mL chloroform/methanol (2:1, v/v) containing 10 mg butylhydroxytoluene per mL of chloroform. The sample solution (100 μL) was allowed to react with 50 μL of DPPP solution (1 mg DPPP dissolved in 10 mL chloroform) in a water bath at 60 °C for 60 min. Then, the reaction mixture was cooled in ice and 3 mL of isopropyl alcohol was added. After diluting the reaction mixture with mobile phase (1:10, v/v), an aliquot of the sample solution was filtered through a 0.45 μm poly tetra fluoroethylene (PTFE) membrane (Acrodisc^®^ Syringe Filters, Pall^®^ Life Sciences, Ann Arbor, MI, USA) and then injected to the HPLC. The HPLC analysis was carried out on a Hitachi L-2350 HPLC system (Hitachi, Tokyo, Japan) with a pump (L-2130), an autosampler (L-2200), and a fluorescence detector (L-2485). The DPPP oxide formed by the reaction of DPPH and lipid hydroperoxides was determined at 40 °C with a Develosil-ODS column (UG-5, Nomura Chem. Co., Seto, Aichi, Japan) protected with an ODS guard column (10 × 4.0 mm i.d.) and the same stationary phase. The mobile phase contained HPLC-grade butanol and methanol (1:9, v/v), and the flow rate was 1 mL/min. The fluorescence detector was set at Ex. 352 nm and Em. 380 nm. The lipid hydroperoxide concentration in the samples was calculated using the DPPP standard curve and was expressed as pmol/g liver.

### 4.6. Analysis of Antioxidant Enzymes and Reduced/Oxidized Glutathione Levels

The activities of SOD and CAT were determined in the liver and eWAT tissue using commercial kits. The levels of GSSG and GSH in both tissues were also quantified using commercial kits. Kits for the estimation of SOD and GSSG/GSH were obtained from Dojindo Molecular Technologies Inc. (Kumamoto, Japan). The CAT kit was purchased from Merck Millipore Corporation (Darmstadt, Germany).

### 4.7. Analysis of Serum Pro-Inflammatory Cytokines Levels

The TNF-α, IL-6, and MCP-1 concentrations in the serum of mice were measured using enzyme-linked immunosorbent assay kits (R&D Systems, Inc., Minneapolis, MN, USA). The serum sample was thawed, appropriately diluted with assay diluents and assayed according to the manufacturer’s protocol.

### 4.8. Total RNA Extraction and Quantitative Real-Time Polymerase Chain Reaction (qRT-PCR)

Total RNA was extracted from the liver and eWAT of mice using the RNeasy Mini Kit (Qiagen, Tokyo, Japan) according to the manufacturer’s protocol. cDNA was then synthesized from total RNA using the High-Capacity cDNA Reverse Transcription Kit (Applied Biosystems Japan Ltd., Tokyo, Japan). qRT-PCR was performed with an automated sequence detection system (ABI 7500, Applied Biosystems Japan Ltd., Tokyo, Japan). The mRNA expression of SOD1, SOD2, CAT, GPX-1, IL-1β, IL-6, MCP-1, TNF-α, and interferon gamma (IFN-γ) were determined with the TaqMan Gene Expression Assays (Applied Biosystems Japan Ltd., Tokyo, Japan). The primers used in this study were as follows: SOD1: Mm01344233_g1; SOD2: Mm01313000_m1; CAT: Mm00437992_m1; GPX-1, Mm00656767_g1; IL-1β: Mm00434228_m1; IL-6: Mm00446190_m1; MCP-1: Mm0041242_m1; TNF-α: Mm00443258_m1; IFN-γ: Mm01168134_m1; glyceraldehyde-3-phosphate dehydrogenase (GAPDH): Mm99999915_g1. The PCR cycling conditions were 50 °C for 2 min, 95 °C for 10 min, and 50 cycles of 95 °C for 15 s followed by 60 °C for 1 min. Data normalization was accomplished using an endogenous reference GAPDH.

### 4.9. Carotenoid Contents in the Liver, Epididymal WAT, and Small Intestine

To confirm the accumulation of carotenoids in the liver, eWAT, and small intestine of mice fed SLC or SLE, total lipids were extracted from these organs or tissue. An aliquot of the extracted lipids dissolved in organic solvent was filtered through a 0.45 μm PTFE membrane prior to HPLC analysis. Then, the HPLC analysis of carotenoids was performed as described above.

### 4.10. Statistical Analysis

Data in the present study were expressed as mean ± standard error (SE). One-way analysis of variance (ANOVA) followed by Tukey’s post hoc test were performed using SPSS software (SPSS Inc., Chicago, IL, USA). Statistical significance was considered at *P* < 0.05. Different letters indicate significant differences among each group.

## 5. Conclusions

Taken together, the present study reveals that supplementation with 4% SLC or SLE for 12 weeks effectively decreased the hepatic lipid hydroperoxide levels as well as increased the activities and mRNA levels of antioxidant enzymes in mice fed a HFHSD. In addition, dietary SLC or SLE also markedly decreased the serum pro-inflammatory cytokines’ levels and down-regulated the mRNA expression of pro-inflammatory cytokines in the liver and epididymal white adipose tissue of mice fed a HFHSD, highlighting the anti-inflammatory action of *Spirulina* lipids. Moreover, the effects of SLC and SLE were comparable. These findings suggest that the antioxidant and anti-inflammatory effects of *Spirulina* lipids may be due to their characteristic lipid compositions and liposoluble components.

## Figures and Tables

**Table 1:** Ingredient composition of experimental diets.

	LFD	HFHSD	SLC	SLE
Ingredient (g/kg)				
Soybean oil	70.00	70.00	30.00	30.00
SLC	-	-	40.00	40.00
SLE	-	-	-	40.00
Lard	-	230.00	230.00	230.00
β-Cornstarch	397.49	110.62	110.62	110.62
α-(dextrinized) Cornstarch	132.00	36.87	36.87	36.87
Casein	200.00	250.00	250.00	250.00
Sucrose	100.00	200.00	200.00	200.00
AIN-93 mineral mixture	35.00	35.00	35.00	35.00
AIN-93 vitamin mixture	10.00	10.00	10.00	10.00
l-Cystine	3.00	3.75	3.75	3.75
Choline bitatrate	2.50	2.50	2.50	2.50
Cellulose	50.00	50.00	50.00	50.00
tert-Butylhydroquinone	0.01	0.06	0.06	0.06
Cholesterol	-	1.20	1.20	1.20

**Table 2:** Fatty acid composition of *Spirulina* lipids and experimental diets.

Fatty acid (wt%)	SLC	SLE	Experimental diets			
			LFD	HFHSD	SLC	SLE
C16:0	46.79	45.69	10.43	18.97	25.66	23.13
C16:1	2.14	2.69	0.11	1.74	2.76	2.55
C18:0	0.71	0.86	4.06	14.90	13.01	13.16
C18:1^†^	2.39	2.62	23.82	46.12	39.38	43.56
C18:2*n*-6	16.78	17.24	53.16	14.76	13.28	13.09
C18:3*n*-3	-	-	5.07	-	-	-
C18:3*n*-6	20.28	22.06	-	-	1.96	2.28

^†^ Including C18:1*n*-9 and C18:1*n*-7.

**Table 3:** Carotenoids contents (mg/g lipids) in *Spirulina* lipids and total lipids extracted from the liver, eWAT, and small intestine of mice fed *Spirulina* lipids.

	*Spirulina* Lipids		Liver		eWAT		Small Intestine	
	SLC	SLE	SLC Group	SLE Group	SLC Group	SLE Group	SLC Group	SLE Group
β-carotene	9.29 ± 0.55	8.57 ± 0.67	^a^ N.D.	N.D.	N.D.	N.D.	0.09 ± 0.02	0.06 ± 0.01
Zeaxanthin	6.18 ± 0.42	8.69 ± 0.86	N.D.	N.D	N.D.	N.D.	0.07 ± 0.02	0.10 ± 0.03

^a^ N.D.: not detected.
